# Multi-Criteria Evaluation of the Failure of CFRP Laminates for Frames in the Automotive Industry

**DOI:** 10.3390/polym14214507

**Published:** 2022-10-25

**Authors:** Ionuț Mititelu, Silviu Mihai Petrișor, Adriana Savin, Roman Šturm, Zoran Bergant, Rozina Steigmann, Mariana Domnica Stanciu, Paul Doru Bârsănescu

**Affiliations:** 1 Faculty of Mechanical Engineering, “Gheorghe Asachi” Technical University, 43 D. Mangeron Blvd., 700050 Iasi, Romania; 2 Nondestructive Testing Department, National Institute of R&D for Technical Physics, 47 D. Mangeron Blvd., 700050 Iasi, Romania; 3 Department of Technical Science, “Nicolae Balcescu” Land Forces Academy, 3-5 Revolutiei Street, 550170 Sibiu, Romania; 4 Faculty of Mechanical Engineering, University of Ljubljana, 6 Aškerčeva, 1000 Ljubljana, Slovenia; 5 Faculty of Mechanical Engineering, Transilvania University of Brasov, 29 Eroilor Blvd., 500360 Brasov, Romania

**Keywords:** CFRP, failure, Arcan device, DMA, stress

## Abstract

Methods to predict the fracture of thin carbon fibre-reinforced polymers (CFRPs) under load are of great interest in the automotive industry. The manufacturing of composites involves a high risk of defect occurrence, and the identification of those that lead to failure increases the functional reliability and decreases costs. The performance of CFRPs can be significantly reduced in assembled structures containing stress concentrators. This paper presents a hybrid experimental–numerical method based on the Tsai–Hill criterion for behavior of thin CFRPs at complex loadings that can emphasize the threshold of stress by tracing the σ-τ envelope. Modified butterfly samples were made for shearing, traction, or shearing-with-traction tests in the weakened section by changing the angle of force application α. ANSYS simulations were used to determine the zones of maximum stress concentration. For thin CFRP samples tested with stacking sequences [0]_8_ and [(45/0)_2_]_s_, the main mechanical characteristics have been determined using a Dynamic Mechanical Analyzer (DMA) and ultrasound tests. A modified Arcan device (AD) was used to generate data in a biaxial stress state, leading to the characterization of the material as a whole. The generated failure envelope allows for the prediction of failure for other combinations of normal and shear stress, depending on the thickness of the laminations, the stacking order, the pretension of the fasteners, and the method used to produce the laminations. The experimental data using AD and the application of the Tsai–Hill criterion serve to the increase the safety of CFRP components.

## 1. Introduction

The automotive industry is known to be the largest consumer of materials, using and integrating products from all branches of modern industry: metallurgy, chemistry, textiles, electronics, etc. In this context, the optimal selection and use of materials is done by taking into account their performance, environmental impact, and cost [1,2,3]. The expectations to extend the lifetimes of cars, to increase external insulation to dampen noise and vibration, and to absorb the kinetic energy of impacts in the event of collisions are just some of the challenges facing the automotive industry [4]. The strategy of reducing weight in order to reduce energy consumption suggests the potential use of composite materials as modular construction elements.

Carbon fibre-reinforced polymers (CFRPs) continue to be used across a wide range of applications, from aerospace systems to automotive, industrial, and consumer goods [5,6]. The high demand for CFRP structures is accompanied by an increase in eco-efficient production performance. The performance of CFRPs can become critical in many engineering applications when changes in temperature, low-energy impacts, or high humidities lead to changes in mechanical properties. Ecological, economic, and operational safety aspects are included in this approach [7].

Composites have evolved as both reinforcements and matrices, with epoxy resin being the most common matrix for CFRPs [8]. Compared to polymer matrices, the properties of epoxy resin are superior (low shrinkage, high environmental durability, high glass transition temperature, low toxicity, etc.), but after ageing, they are susceptible to moisture absorption due to their polar hydroxyl group [9,10]. Composites, which are expected to replace traditional materials to a large extent, have some shortcomings in terms of performance, production and processing procedures, structural complexity, and cost [11,12]. Currently, the classification of composite defects and the identification of damages is a time-consuming and difficult process. It is known that the probability of defects appearing during the manufacturing of composites is still high. Molding processes and component materials such as carbon fibres (CF) lead to the appearance of residual stresses, voids, or excess resins [13]. Together with the transformations that CF undergoes in the matrix (i.e., fiber misalignment), the possible changes in physical properties (module of elasticity, compression, etc.) influence overall mechanical properties such as tension and com-pression [14]. Moreover, processing defects (i.e., drilling and cutting) can develop crack-initiation sites that lead to delamination and premature failure of automotive frame components.

The fibre-reinforced plastic (FRP) does not fail because the fibres reaching their tensile strength limit, but rather because a fracture might develop either in the matrix or at the matrix–fibre interface. The failure of the composite under load, as well the impact damage mechanism of epoxy composite structures, has been investigated in numerous studies [15,16,17]. One way to evaluate the failure load is to strengthen the bond between the fibres and supports by knowing the behaviour of these materials under load and testing different arrangements of fibre stacking. The development of undetectable defects that may affect the structure under the load is tolerated as long as it does not exceed structure’s threshold. Defects and the effects of their development in composites can be classified into two groups: manufacturing defects and accidental service defects. Although using new and advanced nondestructive evaluation (NDE) methods means that the probability of detection (POD) of defects [18,19] is high, this does not exclude the possibility of hidden defects developing during CFRP manufacturing and processing. In the automatic evaluation of the information obtained during the CFRP test [20], knowledge of network and wavelet transformation is required if the automatic segmentation of the images and the 3D reconstruction of the images [21] are applied.

A review of failure mechanisms for FRP composites, including predictions of damages, their evolution, and different Tsai–Hill criteria [22], is available in the literature, in articles such as those written by Hoffmann [23], Chamis [24], and those who developed the Tsai–Wu criterion [25].

Automotive engineers are constantly developing new ways to improve car safety. Gradually, from the point of view of the reinforcements, there was a shift from CF unidirectional (UD) to CF with laminae of different fabrics, improving the formability of the composite. It is already known that the electrical properties depend on the type and volume of CF, which has an average electrical conductivity in the transverse direction between 10 S/m and 10^2^ S/m, with 5 × 10^3^ ÷ 5 × 10^4^ S/m in the longitudinal direction (depending on the type of used CF and their volume fraction in the material), and a relative magnetic permeability is 1 [26]. CF is ideal for the automotive field because it also has a low coefficient of thermal expansion [27]. Repeated exposure to high temperatures was not found to alter this property, which gradually led to the replacement of metal accessories with CF. High formability leads to lower assembly costs due to fewer parts and fasteners, a high specific modulus (modulus/density ratio), and a low coefficient of thermal expansion [28]. Advanced composites are not without integrity issues, as multiple materials in the composition are quasi-isotropic, and the failure mode of the final product could combine failure modes [18]. The high-capacity design leads to an increase in the complexity of the internal geometry and thus to the possibility of discontinuities associated with a flaw [29]. In a laminated CFRP, matrix delamination, cracking, voids, and porosity, in combination with fibre breakage, curling, and bunching, can lead to structural damage [30]. Special attention was paid to the analysis of degradations caused by impact with various energies [31] or overloading of the structure [32]. The damage mechanisms and the effects of non-homogeneity are much more complex in composites compared to metals, which is due to the heterogeneous nature of composites [33]. The formation of cracks due to fatigue loads on the joint components of composites, in adhesives, or in joint areas is often undiagnosed, which leads to large losses [34]. The spatial arrangement, as well as the size of the product, can sometimes save the composite structure from damage.

Investigating the behavior of composites under load, outside the assembly plane, is useful for understanding how it affects the load-bearing capacity of heterogeneous complex structures. Duplication through numerical modeling is helpful for understanding investigations based on experimental tests [28,35], and thus offers a better knowledge for frame design in the automotive industry [36].

It is well known that mechanical fasteners lead to a reduction in the load-bearing capacity of composite structures, due to stresses around the fastener and to the disruption of the continuity of the fibres caused by the drilling of composite panels. The stress distribution at the edge of the fasteners holes is highly dependent on several parameters such as material properties, laminate structure, ply orientation, etc. In addition, specimens are subjected to high-stress concentrators during shear testing, which induce a mixed unknown stress mode that varies along the joints and leads to erratic results [37].

In hybrid joint structures, such as frames in the automotive industry, there are numerous locations that contain stress concentrators, which, together with the detrimental edge effect, reduce the resistance to mechanical loads. Quantitative–qualitative analysis (using FEA and experimental results) of the composite structures that go into the design of complex components can limit undesirable effects. CFRPs are not used only to make lighter and safer cars, but also to replace the car chassis components, lowering the center of the gravity to obtain a vehicle that is more stable in motion and less prone to rollovers. Thin CFRPs offer increased durability and weight reduction for cars without compromising their strength.

Components of the frames carry both static loads and dynamic cycles. When the frame’s geometry is complex, these loads can generate mixed (tension and shear) loading states that can lead to apparition and growth of cracks. Composite damages under mixed loads can be avoided through the prediction of tensile states distribution, analyzing the influence of the internal structure of the sample by material parameter values. To design a composite with high safety for automotive engineering and aircraft construction, in addition to efficient software simulations, experimental assessment is required. These complementary methods assure the identification of weaknesses during design, with maximum safety deployment.

The paper proposes the use of the modified Arcan device for testing thin CFRPs produced using the autoclave method with the [0]_8_ and [(45/0)_2_]_s_ ply-sequences of epoxy/carbon woven laminates. In the multi-criteria evaluation method, the ultrasonic methods are used to explore the propagation of ultrasonic waves through the CFRP to obtain elastic constants, as described in our previous study [38]. In addition, the Dynamic Mechanical Analyzer (DMA) was used to determine the viscoelastic properties such as the temperature-dependent complex modulus and the damping. The multi-criteria evaluation allows for the correlating of mechanical properties under combined and multi-axial loading conditions.

The fracture theory is expressed in terms of various fracture criteria, which have been validated for each specified material, and are functions of stresses that can describe the failure of the material. A prediction method based on the Tsai–Hill criterion is proposed for the behavior of thin CFRPs under the glass transition temperature in complex loading. Since there is no generally accepted fracture criterion worldwide, the determination of the degradation of the structure due to material fracture has to be investigated. The novelty is that the allowable limits of stresses generated by the considered loads are emphasized, facilitating the determination of the load level at which material failure occurs.

## 2. Materials and Methods

### 2.1. Materials and Samples

The nature of the constituents of the material, the volume fraction of fibres, and the sequence and orientation of the lamellae are factors that influence the mechanical and elastic properties of fibre-reinforced composites. The elastic properties of a composite material can be estimated by calculation of relationships, but their exact determination can only be done experimentally [8,13]. In the composite studied, the matrix is polymeric, and the adhesion between the fibres and the matrix results in a structure that is resistant until the fibres break. The manufacturing techniques have shown that these properties depend on the technology used [39,40]. The wet lay-up with vacuum bagging (WLV) is a widely used technology with a low cost, low production rate, and a quality that depends on the skills of the workforce. Therefore, it is very difficult to maintain the uniformity of the surface, which has also been reduced [41,42].

The proposed model uses structure–property–efficiency–cost links. Scientific information and machine learning can facilitate the potential for the preparation and further processing of materials by extracting and identifying valuable information on the materials that will be designed and made.

**Figure 1:**
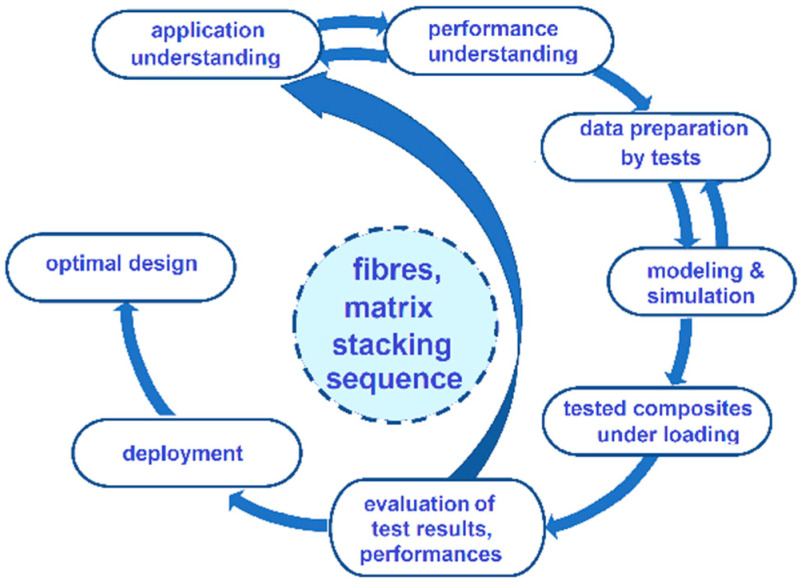
Flowchart of composite assessment.

The model proposed for the study of CFRP performance implies a procedure of simple iterative optimization, in which the analysis of autoclave technology (AT) to identify the optimal mechanical parameters is carried out (verification of the process with the lowest risk at loading), as shown in Figure 1.

Thin CFRP panels with stacking sequences [0]_8_ and [(45/0)_2_]_s_ were realized using AT.

In [43], the method of obtaining CFRP laminates was presented, and was replicated for this study. The samples studied were realized from 4 plates of CFRP, 2 with stacking sequences [0]_8_ (cropping sample S1.1 and S1.2) and 2 for [(45/0)_2_]_s_, (cropping sample S2.1 and S2.2).

CFRP plates were produced by dry preforming with vacuum infusion and curing in an autoclave. CRFP plates were made from 8 layers of plain-weave fabric GG200P and PAN type Pyrofil TR30S 3 K fibers in different ply-stacking sequences, [0]_8_ and [(45/0)_2_]_s,_ respectively. The tensile strength of the filament is 4410 MPa, the modulus is 235 GPa, the density is 1.79 g/cm^3^, the areal weight is 193 g/m^2^, the filament diameter is 6.8 μm, and the coefficient of thermal expansion is 0.5 × 10^−6^ K^−1^. The epoxy resin Epikote MGS LR 285 with slow hardener Epikure MGS LH 287, having a tensile strength 70–80 MPa, a modulus 3–3.3 GPa, and an elongation at break 5–6.5%, was used as matrix.

The dimensions of the plates were 295 × 205 mm^2^ with different thicknesses *d*. The rule of mixture [44] states that the elastic modulus depends on the volume content of fibres V_f_. For the stacking sequences [0]_8_ the volume content value is 0.47 ± 0.003; for [(45/0)_2_]_s_ it is 0.46 ± 0.003, as determined according to the recommendations of ASTM D2584 [45].

**Figure 2:**
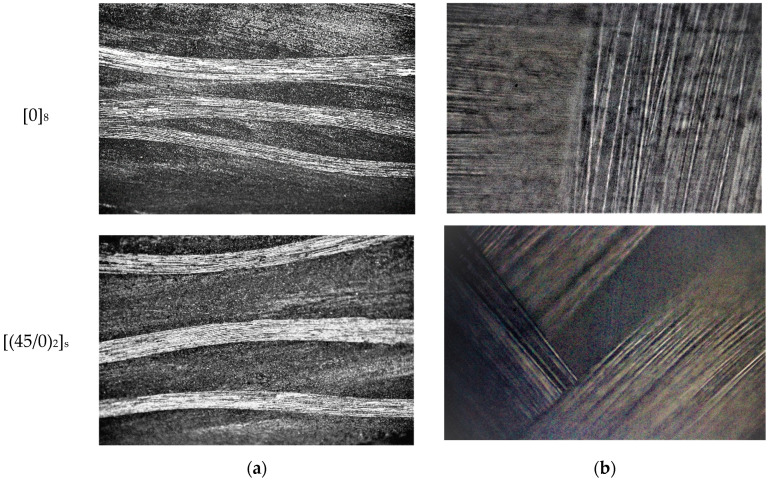
Studied samples: (**a**) cross-sectional view—optical microscope 10×; (**b**) 40× front surface.

The CFRP fabric is plain wave with the warp and weft aligned to form a simple repeating pattern. The properties of the wavy fabric’s structure are analyzed along with the fabric of unidirectional straight threads [46], Figure 2.

The thicknesses of the individual laminae, as well as of the laminated composite, are much smaller than the length or width, such that the planar stress condition can be used to analyze the breaking criterion.

**Figure 3:**
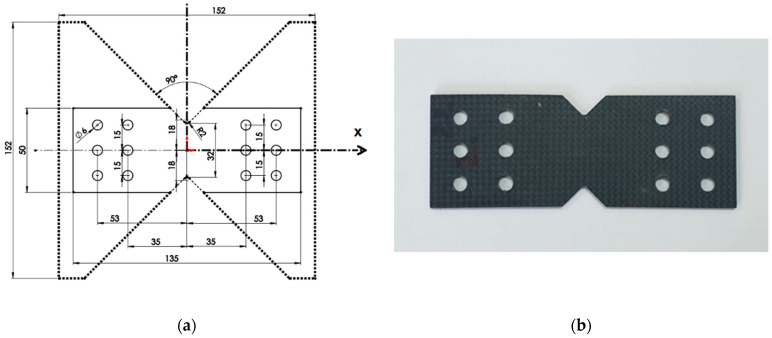
Specimen: (**a**) butterfly sample—scheme; (**b**) modified sample—photo.

The geometry of the specimens was optimized to perform tests to determine the properties of laminates that are subjected to large deformations and the distribution of stress concentrations in the central area. The Iosipescu shear test [47] works well for isotropic materials, while the development of the method for orthotropic materials, particularly composites, is still being studied [48]. Test specimens were made from the 4 plates (using the Iosipescu test specimen model) with flat geometry, each having two symmetrical V-notches (butterfly sample) [47]. The specimen model was used for pure shear and is standardized for different metals, including composites, as shown Figure 3.

The dimensions of the specimens and the notches are chosen so that the tangential stress is as uniform as possible in the central section. The average tangential stress of the central section is $\tau_{1,2}=F/lh$ where *l* is the width of the sample in the section of interest, h the thickness of the sample, and F is the force applied in the test. The design of the butterfly sample was made for testing composites on a modified Arcan device (AD) [49].

The geometry of CFRP specimens for tensile and compressive tests, the methods, and the test conditions are similar to those mentioned in [43]. The data presented in Table 1 are used for simulation using ANSYS workbench to determine the areas of maximum stress concentration for laminate.

### 2.2. Finite Element Analysis (FEA)

**Figure 4:**
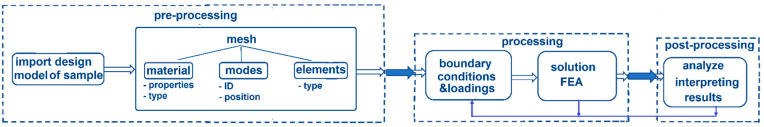
The diagram of FEA.

FEA was performed using a 3D graphic model designed in SolidWorks 2021 and imported into Ansys 2021 R1. In FEA, the fixing of the specimen in AD followed the modelling of the maximum stress distribution in the significant narrow section of the sample, the dependence of the fabric structure on the axis of the applied load, and the influence of the radius of the tip of the notch on the stress concentration. FEA simulations were performed for each type of sample stacking, [0]_8_ and [(45/0)_2_]_s_, with the direction of the fibers being an important parameter. The steps using ANSYS software FEM analysis are shown in Figure 4.

The stresses in the breaking section were calculated using the equations

(1) $$\sigma_{xx}=\frac{F}{A_{0}}\cos\alpha ;\tau_{xy}=\frac{F}{A_{0}}\sin\alpha$$

where *A*_0_ is the narrow section and *α* is the angle between the force direction and the geometric axis of the specimen.

**Figure 5:**
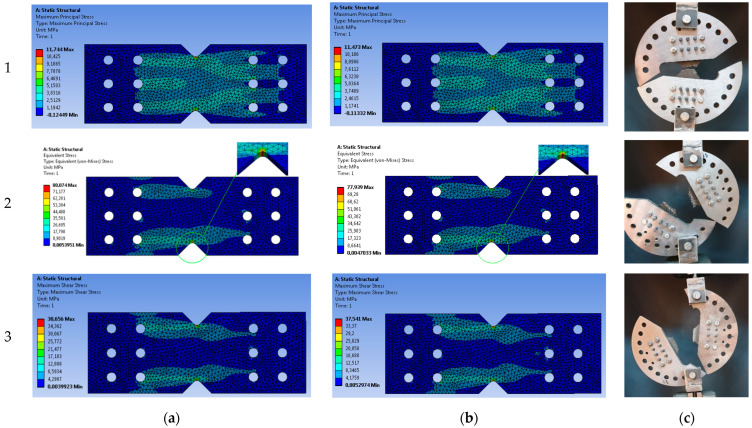
FEA for modified specimen with tip curvature radius 2.54 mm obtained AT in tension (**1**), compound stress in tension with shear *α* = 45° (**2**) and shear (**3**): (**a**) stacking sequence [0]_8_; (**b**) stacking sequence [(45/0)_2_]_s_; (**c**) failed specimens.

The simulations have been carried out without taking into consideration any fiber–matrix microdebonding, which can appear during the tests [50]. The simulation followed the influence on the internal structure of a sample through material parameter values on the stress distribution in the central section. The central area is the one where the most uniform stress is achieved [51]. This conclusion was obtained using FEA for the area in which the notch has a radius of 2.54 mm. The modified butterfly specimen has two notches in the middle, each of 90° and with a depth of 26% of the specimen. The geometry of the specimen was selected for positioning in the device made for testing at different loadings. Figure 5 shows the results of the simulations with the maximum stress zone for the fixed sample under the conditions in which the model transferred to the software has the dimensions from Figure 3 as well as the images of failed specimens to validate their damage with FEA results. The FEA mesh contains 32,048 nodes and 18,429 elements for the sample; the discretization was carried out with tetrahedrons, “FINE” precision, and “uniform” size function.

For biaxial stresses, the stress distribution is still far from the desired uniform distribution. Around the tip of the notch, at a decrease of 0.54 mm of the radius of curvature, the relative variation of the maximum increases by 7.53% for [0]_8_ and 7.35% for [(45/0)_2_]_s_ compared to the radius of 2.54 mm. A smaller radius of the tip of the does not lead to a significant change in the stress distribution. The stress contour in the notch area is shown for biaxial stress. In principle, no significant effect of the fibre orientation angle on the stress distribution can be observed.

### 2.3. Fixture Test

**Figure 6:**
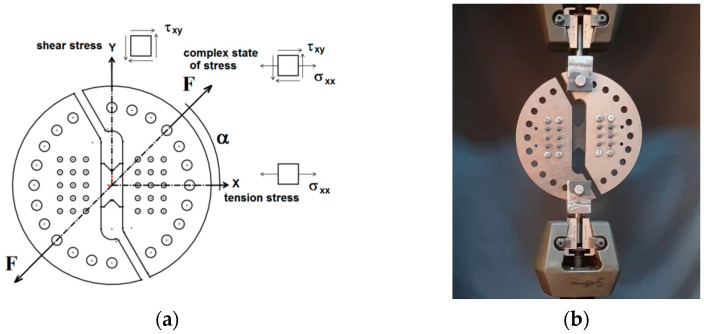
Arcan fixtures for shear test: (**a**) scheme; (**b**) photo.

CFRP butterfly samples were made for AD testing to loadings for shearing, traction, or shearing with traction (with different ratios between σ and τ) in the weakened section by changing the angle of force application α, Figure 6.

The optimization of the design of the proposed device, based on simulations with FE and the experimental results obtained, completes the analysis regarding the performances of the composite materials. The geometry of the samples is selected for positioning in the device to apply the loads. To evaluate the effect of the grip geometry, the distances between the central part and the centre of the two rows of parallel holes, Figure 3a, made on one side and the other of the central area, were determined. The component parts of the Arcan device and the samples are assembled using two-dozen pins (which pass through holes from a sample and from a semi-disk without allowing relative movement) and eight screws, as shown in Figure 6b. During the tests, it was found that the effect of the clamping geometry, the fixing distances from the central part, and the clamping in the support are important. The device allows for the realization of different biaxial stress states (uniaxial stress and shear). AD allows for rotating of the sample by 15° increments in the interval [0÷90°] to produce different biaxial stress states, from traction, to traction with shear, and then up to shear. The samples cropped from the four plates were tested for values of the angle α [30°, 45°, 60°, and 90°]. The modified Arcan samples are fixed on two steel half-disks with two asymmetrical cuts. The shape of the sample concentrates the stress towards its centre, as shown by the cross-section in Figure 6b. All mechanical tests with AD were performed in a WDW Tensile Machine, with a maximum load of 50 kN, and were used to collect data regarding the strength of materials at tensile, shear, and in different biaxial states of stress. The loading applied to the specimens was carried out at a transversal speed of 2 mm/min, and results obtained using AD are presented in Table 2, (in this table the stresses σ and τ were obtained for *α* = 45°).

## 3. Results and Discussions

### 3.1. Experimental Results

Mechanical properties can be used in the design and construction of structures to prevent failure or the presence of areas of potential discontinuities. To this end, non-destructive and destructive evaluation tests have been carried out to analyse the continuous CF laminates and determine the optimal method to obtain them.

#### 3.1.1. Ultrasound Evaluation

**Figure 7:**
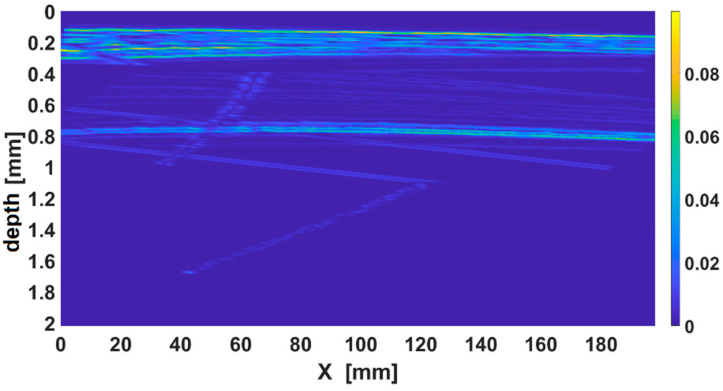
The B scan image of a region of composite with porosity.

The degree of porosity was determined by the US method, using Phasor XS coupled with a phased array with 32 sensors with a pitch of 0.5 mm, a central frequency 5 MHz, and a delay line and is presented in Figure 7. The degree of porosity is linearly correlated with attenuation and with the mechanical properties, i.e. shear resistance. A lower percentage of 3–5% was detected, which is still acceptable [52,53].

The average US propagation speeds in the material were determined [54] to be C_l_ = 2754 ± 20 m/s and C_t_ = 1945 ± 20 m/s for samples [0]_8_, and to be C_l_ = 2840 ± 20 m/s and C_t_ = 1970 ± 20 m/s for samples [(45/0)_2_]_s_. The values represent the average of 50 determinations at different points of the samples, the dispersion being calculated with the standard method [55]. The Poisson coefficient was also determined to be ν_xz_ = ν_yz_ = 0.3 using this value of velocity.

#### 3.1.2. DMA Analyses

The main mechanical characteristics of studied samples with two types of stacking sequence have been determined using Dynamic Mechanical Analyzer, DMA 242C—Netzsch Germany, with the 3-point bending device using Protheus software v.4.8.5. The parameters obtained from the tests provide information on vitrification, referred to T_g_ (glass transition), resulting from the cross-linking reaction. The determinations were made at only 2 frequencies, 1 and 5 Hz, at room-controlled temperature, to obtain the apparent activation energy for the glass transition process. Samples with dimensions 50 × 10 × 1.98 [mm] were cut from the composite plates in stacking sequences [0]_8_ and [(45/0)_2_]_s_. The mechanisms for changing the elastic and viscoelastic storage modulus (*E′*), mechanical properties (*δ*), and glass transition (T_g_) were analyzed.

The ratio of stress (σ) to strain (*ε*) under dynamic load is defined as the complex Young modulus of the material (E*) which can be expressed as

(2) $$\begin{array}{l} E^{*}=\frac{\bar{\sigma }}{\bar{\varepsilon }}=\frac{\sigma_{0}}{\varepsilon_{0}}e^{i\delta }=E^{\prime }+iE^{\prime \prime }\\ \tan\delta =\frac{E^{\prime \prime }}{E^{\prime }} \end{array}$$

**Figure 8:**
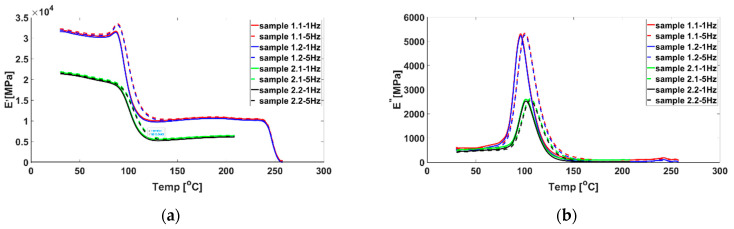

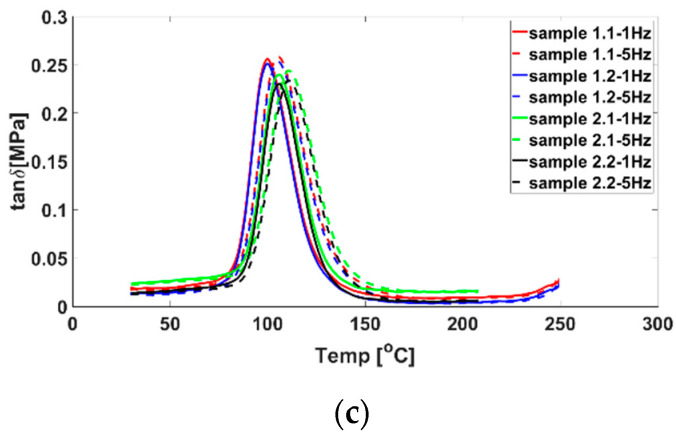
DMA results for samples 1.1 and 1.2 from plates with stacking sequence [0]_8_; and samples 2.1 and 2.2 from plates with stacking sequence [(45/0)_2_]_s_: (**a**) *E′*; (**b**) *E″*; (**c**) tan *δ*.

The DMA analysis of the CFRP plates made in the two stacking modes shows that the changes in the dynamic mode of the materials under the vibration load with temperature do not differ significantly. The loss modulus reflects the adhesion of the material. The storage modulus, i.e., the modulus of elasticity reflects the stiffness of the material. The ratio between the loss and storage modules is the damping of the material tan *δ. δ* is the phase angle of stress and strain. The maximum value of tan *δ* is close to the glass transition temperature of CFRPs. The graph shows that the storage modulus (*E′*) increases slightly with increasing frequency at low temperatures, as seen in Figure 8. It is observed that the strength of the material is satisfactory up to about 97 °C for both types of stacking, after which it decreases in the interval [90–150 °C]. Under the conditions in this interval, the secondary hardening of CFRPs takes place as a result of the reaction between the functional groups of the resins.

It can be seen from Figure 8 that the modulus of elasticity of the epoxy resin decreased rapidly when the temperature increased from 98 ÷ 117 °C. The loss modulus fluctuated violently when the temperature increased from 90 ÷ 120 °C, indicating that the temperature significantly influenced the adhesion activity of the resin. The properties of the material changed at this temperature, as the material passed from a glassy state and entered the “high elastic” zone, where the viscoelastic properties of the polymer are more pronounced.

The value of *E′* decreases slowly around the temperature values included in the interval [92÷97] °C for both types of stacking when a transition temperature T_g_ to the glassy state is reached, according to Figure 8a,b, respectively. Interpreting the results obtained from the DMA tests, it can be stated that the glass transition is reversible for both types of stacking, up to the temperature at which tan (*δ*) reaches its maximum (96.85 °C) for CF/Epoxy [0]_8_, respectively 97 °C for CF /Epoxy [(45/0)_2_)]_8_. T_g_ represents a main parameter related to the heat resistance of the resin, which is why T_g_ is considered the critical upper limit taken into account in the design and operation of composites. Near T_g_, the physicochemical properties and failure stress during bending change significantly.

**Figure 9:**
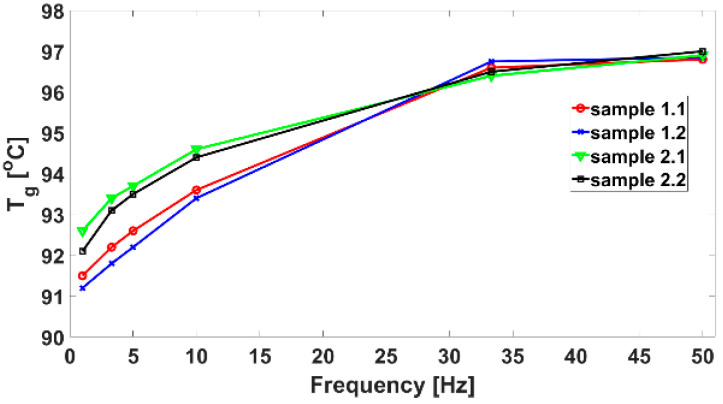
Glass transition temperature for the 4 samples taken into study.

From dynamic test modes with CFRPs, it is observed that with the increase of the vibration frequency, there is an increase in T_g_ by one degree for the stacking sequence of [(45/0)_2_)]_s_ compared to [0]_8_, as seen in Figure 9. The phenomenon is normal, and is related to the viscoelastic behaviour of the resin. Fibre orientation appears to reduce the range of variation of T_g_ from 5.3 °C for [0]_8_ to 4.9 °C for [(45/0)_2_)]_s_ and therefore increases T_g_ as the upper critical safe-design limit.

### 3.2. Failure Mechanism Criteria

The failure of the material occurs as a loss of the ability to withstand stress higher than a limit value. Due to the lack of a universally accepted breaking criterion, the determination of the degradation of the structure due to material breakage is still extensively studied [56]. The strength of a layered composite is determined by the strength of the component laminae, their orientation, and their stacking sequences. Analytically, the fracture theory is expressed in the form of different fracture criteria, validated for each specified material, and is a function of stresses that can describe material’s failure. At the microscopic level, breaking represents the initiation and propagation of a crack; at the macroscopic level, material breaking is defined by the load capacity or energy storage capacity. The Tsai–Hill failure criterion [57] is based on the von Mises criterion (strain energy theory of shape variation) for the plasticity of metals. Initially extended to anisotropic materials by Hill [22], Tsai–Hill is one of the widely used criterions for composite materials [56]. For a particular biaxial state of stress with uniaxial tension and shear, the Tsai–Hill equation will determine only the normal stress σ_11_ (σ_22_ = 0) and where the rupture will occur. This for a biaxial state of stress becomes

(3) $${\left(\frac{\sigma_{11}}{X_{11}}\right)}^{2}+{\left(\frac{\tau_{12}}{X_{12}}\right)}^{2}=1$$

where *X*_11_ is the tensile strength in the longitudinal direction; *X*_12_ is plane shear strength. The criterion chosen is an interactive criterion, meaning that the stresses from different directions are not decoupled, thus affecting the simultaneous failure, independent of the failure type (as it does not estimate the type of failure). The Tsai–Hill breaking theory has the advantage that it is expressed through a unique criterion; by applying it, the interaction between the components of the main stresses is taken into account to an appreciable extent *σ*_11_, *σ*_22_, and *τ*_12_, so that the estimation results are closer to the experimental data. Equation (3) represents an ellipse in space *σ*-*τ*, with the major and minor semi-axes *X*_11_ and *X*_12_. In this paper, the values for *X*_11_ and for *X*_12_ in shear were determined experimentally through tensile tests with dog bone specimens (by the Iosipescu method, for example).

In coordinates *σ-τ* on the contour of the ellipse, the material reaches the admissible limit, the safety factor being *c* = 1. For the situations in which *c* > 1 or *c* < 1, the space is divided into the safe zone inside the ellipse, with the dangerous zone outside. The safety coefficient *c*_1_ in the case of the criterion is calculated from the relationship

(4) $$c_{1}^{2}\left[{\left(\frac{\sigma }{X_{11}}\right)}^{2}+{\left(\frac{\tau }{X_{12}}\right)}^{2}\right]=1$$

where *σ* and *τ* are the stress components calculated for an applied load at a certain *α* angle.

**Figure 10:**
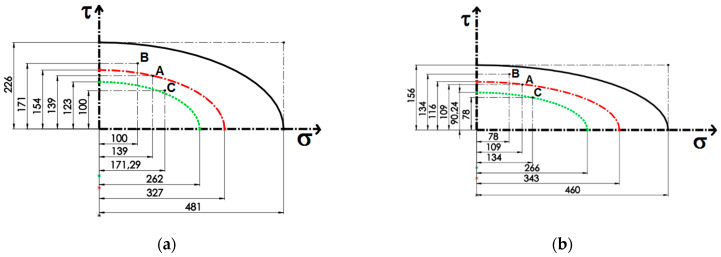

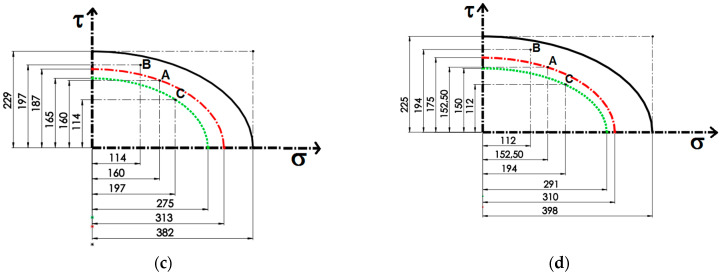
Prediction from maximum Tsai–Hill failure criteria for composite structures: (**a**,**b**) with stacking sequence [0]_8_ and (**c**,**d**) stacking sequence [(45/0)_2_]_s_.

Figure 10 shows the ellipses generated using the Tsai–Hill criterion. These will be traced knowing the ellipses semi-axes, respective tensile strength *X*_11_, and experimentally determined shear strength *X*_12_.

For each diagram, three points (experimentally determined) are represented, marking the failure of the composite material: point A for the loading direction at 45°, point B for the loading direction at 60°, and point C along the loading direction at 30°.

If, under certain loading conditions, the failure of material takes place in the safety-relevant area, there are serious design problems.

The engineering calculation problem is solved by tracing a new concentrical ellipse with semi-axes proportional to first ellipse (the continuous line) which delimits the new safety zone. The semi-axes of this ellipse are *X*_11_/*c*_1_ and *X*_12_/*c*_1_, respectively, where *c*_1_ is a factor of safety (*c*_1_ > 1). So, for engineering design, the states of stress that produce the failure of the material will be considered on the contour of the smaller ellipse or outside of it. Thus, the safe area will now be considered as being inside smaller ellipses. In Table 3, the safety factor c_1_ is presented for the four samples studied.

The safety factor *c*_1_ has been chosen so that the C point shall not belong to the safety zone. Of course, in the engineering calculation, *c*_1_ might be considered minimal because the material fails in point C.

Choosing a failure criterion is not an easy task, because no criterion is universal. The chosen criterion can lead either to good or to simply satisfactory results depending on the load, and the stacking structure of the composite material, and the polymer resin.

For the case of applying the force at an angle of 30°, the composite material still presents the failure risk, so a new safety coefficient *c*_2_, for which the envelope clearly delimits the safety zone, is presented in Table 4.

These types of plots and the ellipses equations established can be used for design, allowing for the determination of a combination between normal stress and shear stress under which the material fails.

## 4. Conclusions

The experimental results using the modified Arcan device and modified butterfly specimens from composite materials with the stacking sequences [(45/0)_2_)]_s_ and [0]_8_, respectively, for which fracture criteria were applied, led to the following conclusions.

Fracture characterization of composite materials cannot be performed with precision using only simple one-run tensile experiments. The use of the Arcan device for testing allows combined tests to generate data at the biaxial stress state using the universal testing machine, leading to the characterization of the material as a whole.

The failure envelope of material is designed through a curve fitting procedure, as some points of the envelope have been determined experimentally. The failure envelope, once traced, can be used for any further combinations of normal stress and shear stress, which is useful in the designing of components. This proves that the fracture’s envelope depends on different physical properties and mechanisms. The safe zone will now be considered as the one inside the small ellipse, and the Tsai–Hill criterion can be easily rewritten for each composite material studied in this work. The obtained results show that the failure criteria taken into consideration are correct and can be used for the prediction of failure in the analyzed composite structures.

The modified Arcan device can be used to characterize the mechanical behaviour of composites below the glass transition temperature for complex loads (tension and shear). The device is mounted on a tensile testing machine and is easy to use. The glass transition temperature of CFRP plates in two stacking sequences was determined to investigate if the stacking mode affects the crosslinking density of the resin and is therefore an additional improvement of the mechanical properties.

In the failure study of CFRP specimens, factors such as the thickness of the lamina, stacking sequences of the laminates, pretension of fixing elements, and the method for obtaining laminates must be taken into account.

The experimental data obtained using AD enable the correct application of the Tsai–Hill criterion and thus contribute to increasing the safety of the components manufactured from the investigated CFRPs. The limit state envelope was drawn for the investigated materials. For this study, the safety factors between 1.067 and 1.46 were used.

The theoretical curves were produced to confirm the experimental results. The results of FEA simulations indicate that the modified specimens proposed here can replace the butterfly specimens, leading to reduction in the consumption of the composite materials and a reduction in the dimensions of AD. Additional tests are required for each CFRP specimen to avoid premature failure of the specimens in the area of the fasteners due to excessive contact pressures.

## Figures and Tables

**Table 1:** Material properties for samples taken into study.

		Stacking Sequence			
		[0]_8_		[(45/0)_2_]_s_	
*d* (mm)		S1.1: 1.98 and S1.2: 2.0		S2.1: 2.0 and S.2.2: 2.04	
V_f_ (%)		45	47	44	46
Tension	E_1_ [GPa]	33			
	X_1_^T^ [MPa]	481	460		
	ε_1_^T^ [%]	0.862			
	E_x_ [GPa]			22.6	
	σ_x_^T^ [MPa]			382	398
	ε_x_^T^ [%]			1.015	
Compression	E_1_ [GPa]	37			
	X_1_^C^ [MPa]	420			
	ε_1_^C^ [%]	0.9			
	E_x_ [GPa]			28.6	
	σ_x_^C^ [MPa]			312	
	ε_x_^C^ [%]			1.08	

**Table 2:** Results of pure-shear and mixed shear/tension ARCAN test: Factor of safety c_1_ (calculated for ellipses drawn with dotted line).

Sample	σ [MPa]	τ [MPa]	X_11_ [MPa]	X_12_ [MPa]
S1.1	139.42	139.42	481	226.325
S1.2	109.59	109.59	460	156
S2.1	160	160	382	229.5
S2.2	158	158	398	225

**Table 3:** The factor of safety *c*_1_.

Sample	The Semi-Axes of the Ellipse Which Delimits the Area Considered Safe		c_1_
	*X*_11_/*c*_1_ [MPa]	*X*_12_/*c*_1_ [MPa]	
S1.1	481/*c*_1_ = 327.43	226/*c*_1_ = 154.06	1.46
S1.2	460/*c*_1_ = 343.28	156/*c*_1_ = 116.41	1.34
S2.1	382/*c*_1_ = 313.11	229/*c*_1_ = 187.70	1.22
S2.2	398/*c*_1_ = 310.93	225/*c*_1_ = 175.78	1.28

**Table 4:** The factor of safety *c*_2_.

Sample	The Semi-Axes of the Ellipse Which Delimits the Area Considered Safe		*c*_2_
	*X*_11_/*c*_2_ [MPa]	*X*_12_/*c*_2_ [MPa]	
S1.1	327.43/*c*_2_ = 262	154.06/*c*_2_ = 123.39	1.248
S1.2	343.28/*c*_2_ = 266	116.41/*c*_2_ = 90.24	1.29
S2.1	313.11/*c*_2_ = 274.65	187.70/*c*_2_ = 164.64	1.14
S2.2	310.93/*c*_2_ = 290.58	175.78/*c*_2_ = 164.28	1.068

## Data Availability

Not applicable.
